# Value of a Chemical and Microscopic Examination of the Urine as a Means of Diagnosis in Renal Diseases

**Published:** 1851-06

**Authors:** 


*Value of a Chemical and Microscopical Examination of the Urine as a means of Diagnosis in Renal Diseases.*—Dr. Geo. Johnson makes the following just remarks on this subject in an interesting paper in the *London Journal of Medicine* (Feb. 1851):—

"None," he says, "but those who have paid much attention to the diseases of the kidneys, and to the means of distinguishing one form of disease from another, can form an estimate of the true value of the information to be derived from a chemical and microscopical examination of the urine. It may safely be affirmed, that a microscopical examination of the urine is, in general, as necessary for the formation of a correct opinion in cases of renal disease, as a physical examination of the chest is for the exact diagnosis of diseases within that cavity. To determine the true nature of disease in the kidney, and to form a probably correct judgment as to its course and termination, it is not sufficient merely to ascertain that albumen is present in the urine, that the specific gravity of the secretion is abnormal, and that moulds of the uriniferous tubes may be detected by the microscope. These signs may each and all be present in a given case; but a more precise examination of the circumstances is necessary for the formation of a correct diagnosis. The mere enumeration of these signs, such as is very commonly given in reporting cases of renal disease, will as little enable a practitioner to ascertain the true nature of disease in the kidney, as would a simple report of a patient having cough, with dyspnœa, expectoration, and a sound produced by the mixture of air and liquid in the air-tubes, suffice for the exact diagnosis of disease in the lung. In both instances, the signs must be exactly observed, and correctly reported; otherwise no sound opinion can be based upon them.

"It is well known that diseases of the kidneys are amongst the most frequent of those which destroy life, and that some forms of renal disease are curable, while others are almost certainly fatal. In many of the most obvious circumstances, all the forms of renal disease are alike; and it is only by a careful attention to differences, which, however minute, may yet be readily and certainly appreciated, that we can hope to distinguish one disease from another, or to give a confident opinion as to the probable course and termination of a case.

"The microscopical examination of the urine is much facilitated by placing it in a conical glass, which will contain about three or four ounces. The fluid should be allowed to stand for a few hours before examination. I usually put it in the glasses at night, and examine it with the microscope on the following morning. This delay is not necessary if the sediment is abundant; but in many cases, when the sediment is small in quantity, it is quite essential that time should be allowed for its deposition to take place. A small quantity of the lower part of the liquid should then be removed with a pipette, and placed in a shallow cell for microscopical examination."—*Am. Jour. Med. Sci.*

